# Assessment of mental workload across cognitive tasks using a passive brain-computer interface based on mean negative theta-band amplitudes

**DOI:** 10.3389/fnrgo.2023.1233722

**Published:** 2023-11-23

**Authors:** Guillermo I. Gallegos Ayala, David Haslacher, Laurens R. Krol, Surjo R. Soekadar, Thorsten O. Zander

**Affiliations:** ^1^Department of Psychiatry and Neurosciences, Clinical Neurotechnology Laboratory, Charité – Universitätsmedizin Berlin, Berlin, Germany; ^2^Neuroadaptive Human-Computer Interaction, Brandenburg University of Technology Cottbus-Senftenberg, Cottbus, Brandenburg, Germany; ^3^Zander Laboratories B.V., Amsterdam, Netherlands

**Keywords:** brain-computer interface, mental workload, passive BCI, classification of neural signals, support vector machine, cognitive task, frontal midline theta, parietal alpha oscillations

## Abstract

Brain-computer interfaces (BCI) can provide real-time and continuous assessments of mental workload in different scenarios, which can subsequently be used to optimize human-computer interaction. However, assessment of mental workload is complicated by the task-dependent nature of the underlying neural signals. Thus, classifiers trained on data from one task do not generalize well to other tasks. Previous attempts at classifying mental workload across different cognitive tasks have therefore only been partially successful. Here we introduce a novel algorithm to extract frontal theta oscillations from electroencephalographic (EEG) recordings of brain activity and show that it can be used to detect mental workload across different cognitive tasks. We use a published data set that investigated subject dependent task transfer, based on Filter Bank Common Spatial Patterns. After testing, our approach enables a binary classification of mental workload with performances of 92.00 and 92.35%, respectively for either low or high workload vs. an initial no workload condition, with significantly better results than those of the previous approach. It, nevertheless, does not perform beyond chance level when comparing high vs. low workload conditions. Also, when an independent component analysis was done first with the data (and before any additional preprocessing procedure), even though we achieved more stable classification results above chance level across all tasks, it did not perform better than the previous approach. These mixed results illustrate that while the proposed algorithm cannot replace previous general-purpose classification methods, it may outperform state-of-the-art algorithms in specific (workload) comparisons.

## 1 Introduction

Passive brain-computer interfaces (pBCIs) based on electroencephalography (EEG) can be leveraged to assess cognitive and affective states of the user in real-time, enabling flexible human-computer interaction (Zander and Kothe, 2011; Mühl et al., 2014; Krol et al., 2018). Ideally, such interaction takes place seamlessly, without the explicit instruction of the user, enabling a form of neuroadaptive technology that does not place any additional burden of control on the user (Zander et al., 2016). For instance, during operation of heavy machinery in a safety-critical environment, additional automation may be required to avoid potentially fatal errors during periods of high workload. A pBCI that can assess mental workload directly from ongoing brain activity could be crucial to predict the occurrence of, and avoid, such errors.

Despite decades of research, there is no consensus among researchers regarding a clear definition of mental workload, and a unified framework for it does not exist in the literature, but in general, it refers to the cognitive effort an individual dedicates to a task. Longo et al. (2022) mention this obvious pre-existence of two interacting entities (a subject and a task) in any definition of workload, but different theoretical assumptions, as well as approaches to it always complicate a final agreement. Workload is a highly complex and multifaceted concept, but a consistent theme among various interpretations is its impact on performance and its prediction. This also lies at the core of its importance in a variety of fields. Both over- and underload negatively affect performance and can lead to dangerous situations. Therefore, it is important to be able to obtain an accurate measure of workload. Currently, mental workload is assessed using psychological approaches such as surveys, or peripheral physiological measures of arousal such as heartrate variability (Fine et al., 2022). However, such indirect measures do not provide the means to assess mental workload in real time, as they are a delayed and indirect measure of cognitive processes. While the different theoretical assumptions related to the different interpretations of workload as a concept bring complications, the use of brain activity can help provide a data-driven measurement. BCIs on the other hand, can detect changes in brain activity directly reflecting mental workload. One such electrophysiological measure is frontal theta activity, which can be assessed to detect increases in mental workload from EEG signals (So et al., 2017). In this work we use this direct approach, by using measurements of brain activity with a BCI.

Since BCIs need to be specifically calibrated to detect the patterns of EEG activity related to a specific person performing to a specific task, the application of BCIs to assess mental workload is commonly restricted to one task at a time (Zhang et al., 2018). In these cases, the classifier is trained and tested on data from the same cognitive task. In contrast, it would be desirable to develop a system which can detect increases in mental workload independently of the task used for calibration (training). This transfer learning problem is known to be challenging for EEG due to the covariate shift (Li et al., 2010). For instance, in the application at hand, the baseline level of frontal theta activity may not be same across tasks, preventing successful generalization of the trained classifier.

Previous work has investigated the possibility of detecting the level of mental workload from EEG signals across different tasks. Krol et al. (2016) initially showed that a filter-bank common spatial patterns classifier could reliably differentiate between high and low workload in a multiplication (69 ± 13% accuracy) and word recognition (76 ± 15% accuracy) task, when trained on a subtraction task specifically designed to enable such transferability (Zander and Krol, 2017). While these results indicated that transfer learning of workload detection across tasks is feasible, the types of tasks and the levels of workloads tested were limited. Therefore, a subsequent study employed the same filter-bank common spatial patterns algorithm to detect both low and high levels of workload in a variety of tasks commonly used in workload research (Zhang et al., 2018). While the transferability was confirmed for some tasks, it was found that no meaningful performance above chance level (Mueller-Putz et al., 2008) could be obtained in other tasks.

In the present study, we developed a novel classification algorithm to detect mental workload based on frontal theta oscillations. Using our algorithm on the dataset published in Zhang et al. (2018), we show that a significant improvement in classification performance can be obtained, providing a feasible method of classifying mental workload across tasks commonly used in the field.

## 2 Methods

### 2.1 Experimental setup and data acquisition

The data analyzed here was originally published in Zhang et al. (2018). Details on data acquisition are reproduced here for completeness. Fifteen participants (28.6 ± 4.1 years of age) performed a series of tasks on a computer with a 27-inch screen in front of them while 64-channel EEG (BrainAmp DC, Brain Products GmbH, Gilching, Germany) was recorded. Participants performed six tasks. A calibration task T0 was always presented first, and consisted of a no workload condition, and a workload condition. Each of the remaining five tasks T1-T5 consisted of two workload conditions (high and low). Here we briefly outline the tasks.

T0, the calibration task, presented an equation in the form of a - b where in the workload condition participants were instructed to keep on subtracting b from the previous result. In the no workload condition, participants were shown a crosshair and instructed to focus their thoughts inwards and relax. In each trial, regardless of condition, there was a 50% chance that 10 visually distracting “sparkles” floating slowly around the screen appeared. These served to elicit and control for eye movement artifacts. Per condition, 20 trials were performed of 10 s each for a total of 200 s per class.

T1 was an N-Back task (Kirchner, 1958), that used a sequence of numbers, where participants had to indicate by a button press whether or not the current number was equal to the Nth previous number. N was equal to 1 and 3 in the low and high load conditions, respectively. Per workload condition, 12 trials were performed each lasting 25 s. The order of the trials was randomized.

T2 was a backward span task, presenting a sequence of numbers one digit at a time. Participants were instructed to memorize the sequence and, at the end of each trial, reproduce it in reverse order. The sequence length was 2 for the low and 6 for the high load conditions. Per workload condition, 10 trials were performed each lasting 30 s. The order of the trials was randomized.

T3 was an arithmetic addition task in which participants were instructed to add two presented numbers, chosen such that the Q-value (Thomas, 1963; Walter et al., 2017) of the resulting addition was between 2 and 2.5 for the low, or between 4 and 5, for the high load conditions. Per workload condition, 10 trials were performed each lasting 30 s the order of the trials was randomized.

T4 was a word recovery task, in which participants were instructed to identify a word whose characters had been mixed in a different order before it was presented on the computer screen. German words from the SUBTLEX-DE corpus (Brysbaert et al., 2011) were used: two-syllabled high-frequency words in low load, and three-syllabled low-frequency words in high load. Per workload condition, 10 trials were performed each lasting 30 s. The order of the trials was randomized.

T5 was a mental rotation task (Shepard and Metzler, 1971), in which participants were instructed to identify whether two figures of rotated objects were the same shape, or mirrored versions of each other. Following (So et al., 2017), the low load condition used two-dimensional shapes consisting of connected squares while the high load condition used three-dimensional shapes consisting of nine connected cubes. Per workload condition, 10 trials were performed each lasting 30 s. The order of the trials was randomized.

EEG data were acquired from 15 subjects using a 64 active electrode EEG system (BrainAmp DC, Brain Products GmbH, Gilching, Germany, using actiCAP electrode caps) organized according to the extended 10- 20 system. Data was sampled at 500 Hz. Due to technical difficulties, some data was missing from three participants.

For the present study, we have considered the full data available from 12 participants. Also, as task execution times were not fully reached in some cases, for uniformity in the treatment along all the subjects and tasks we cut-off with the following timeframe durations (starting at the beginning of each task and workload condition) in all our analyses: T0: 9 s, T1: 20 s, T2: 24 s, T3-T5: 30 s.

### 2.2 Preprocessing

Previous research has indicated that an increase in magnitude of frontal theta oscillations relates to an increase in working memory load levels (Gevins et al., 1997; Jensen and Tesche, 2002; Gerjets et al., 2014), and mental workload (So et al., 2017). Missonnier et al. (2006) found a frontal theta event related synchronization (ERS) of higher amplitude in a 1-back, a 2-back, and a detection task as compared to a passive fixation task. Therefore, we first bandpass-filtered EEG data into the theta (4–7 Hz) band using a 5th order Butterworth filter. Next, parameters for trimming, scaling, and channel extraction were optimized on the data.

**Figure 1:**
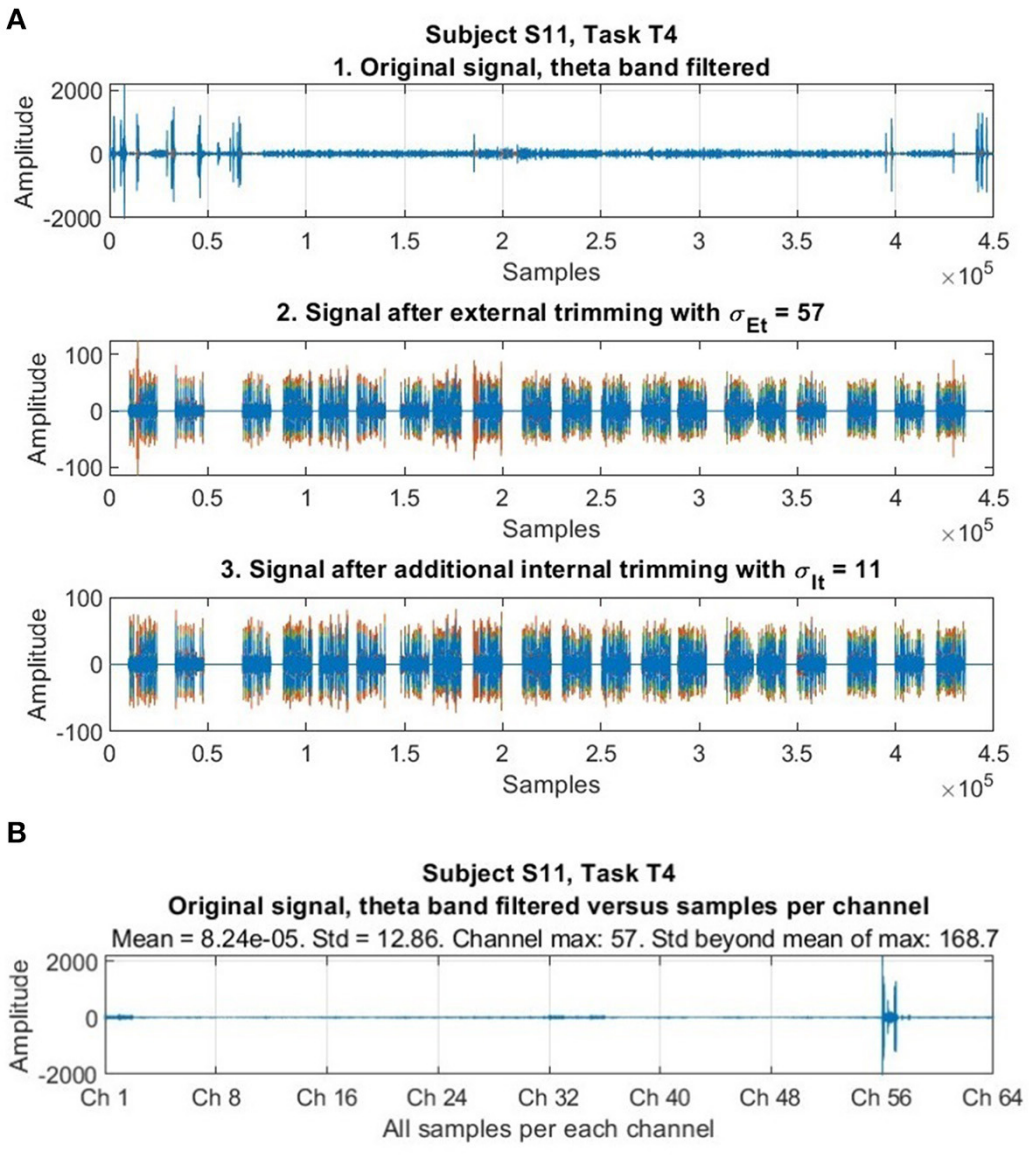
Illustration of the trimming procedure. **(A)** The first stage shows an original signal after filtering through the theta band contaminated with high aberrant values (abnormal and not representative as to be considered to do meaningful comparisons and scaling, and whose effect should be neutralized, reduced, or removed). The second stage shows the effects of inter-channel trimming (with σ_Et_ = 57), that already accomplishes its purpose of neutralizing these amplitude values from the time series while preserving the rest of the signal. The third stage shows the effect of intra-channel trimming (with σ_It_ = 11), which is very fine and preserves the signal very well. **(B)** The same original EEG signal with sequential samples organized per channel (along all the 64 EEG channels) in the horizontal axis, and the amplitude of samples in the vertical axis. High aberrant values are not dispersed or “distributed” throughout the whole signal, but rather clustered or concentrated in isolated channels, a particularity corroborated in our whole dataset after throughout scrutiny. Here, it is revealed that aberrant values going up to 165.8 standard deviations beyond the mean exist, but they are concentrated in Ch 57 only. Amplitudes correspond to voltages in microvolts (μV).

We employed a trimming procedure to remove abnormally large voltage values reflecting artifacts, similar to Delorme and Makeig (2004). Samples exceeding a fixed threshold of standard deviations below or above the mean across space and time were removed. Because abnormal, extreme values in datasets -often called “outliers”- pose a problem in statistical data analysis (Barnett, 1979), and EEG signals “typically are contaminated by measurement artifacts and noise from non-neurophysiological sources” (Dornhege et al., 2007), researchers have proposed solutions to identify these atypical values, neutralize or reduce their effect, or eliminate regions of data containing them, as in EEG channel suppression (e.g., Birch et al., 1993; Tax and Duin, 2002; Harmeling et al., 2006). According to Duda et al. (2001) the trimmed mean of a distribution is less sensitive to the presence of outliers than is the sample mean. We performed an external (or inter-channel) trimming of samples whose voltage amplitude was σ_Et_ = 57 standard deviations beyond the mean of each specific dataset considered -which was equally done for all the data, where each specific set consisted of the data of all the 64 EEG channels for one subject, and task. This procedure was executed once per each set. We performed an internal (or intra-channel) trimming individually (or internally) for each channel in each particular dataset (consisting of the data of one subject and task). The internal trimming procedure was carried out iteratively while samples remained whose voltage amplitude was σ_It_ = 11 standard deviations beyond the mean. In each internal trimming iteration, the mean of the dataset was updated (as did the highest and lowest amplitude values of the corresponding samples also change). Figure 1 illustrates, among many trials available, the principle of trimming with the EEG signal of a specific subject and task.

The trimming parameters were optimized and selected after an exhaustive observation of all the data. It was noted -as in Figure 1- that high aberrant values were concentrated in only a few channels (out of the total 64) in some parts of the data. Our trimming procedure was applied to treat all data equally and automatically, after finding suitable trimming parameters that first, effectively neutralize extreme abnormal values, and second are optimized and chosen so that, at the same time, they do not affect original data that should not change. We tested several combinations of candidate values to control this requirement for each dataset (belonging to one subject and task). After evaluation, once suitable parameters for our dataset were found, trimming was applied equally to all the data, but its application only affected an estimated 1.63% of the data (a 98.37% of the data remained intact).

One of our guiding objectives (in this study and beyond) is the implementation of universal classifiers of workload, that is, a successful implementation of task, subject and session transfer. One problem, according to the existing literature, are the differences between the conditions of each recording session. As we implement classifiers able to switch between subjects, tasks, and sessions, models of data able to deal and compare the heterogeneity of trials recorded in different measurements, from different subjects are needed. For this, we use scaling which is a preprocessing procedure consolidated in the literature of pattern recognition. For instance, Rowley et al. (1998) implemented scaling in face detection -because in original pictures faces to be recognized might have different sizes, but automated classifiers need coherent parameters (i.e., concrete 20 by 20 pixels image windows)- together with specific preprocessing techniques (such as light correction, and histogram equalization of pixels of original images, before any classification procedure could be used, because images in general are also recorded from a variety of “measurements,” with different input gains). As in this case, specific problems might require specific approaches for a solution, and when the literature in a particular area of knowledge reports the existence of a problem, and after a while it continues reporting the same problem it is likely that the existing methods are not able to bring satisfactory solutions. We realized the importance of scaling for universal implementations of classifiers of brain activity, but as existing approaches to EEG signal preprocessing (such as standard min-max scaling) have not let the research community hear about a solution it might be the right time to try variations, new approaches, and/or complementary techniques that might well contribute to it. We scaled the data from different subjects and measurement sessions to a single universal scaling factor each time, to unify and perform meaningful comparisons. Universal scaling factors are arbitrary, external, and independent from the data. Each subset that is scaled represents the mental activity of one subject executing one of the tasks. In addition our approach to scaling (in opposition to standard min-max scaling) also takes the positive and negative components of signals separately in consideration to previous studies (Jones, 2016) which sustain that traditional preprocessing approaches, such as band-pass filtering and detrending, fit signals and impose them with the appearance of perfectly symmetric sinusoidal waves that oscillate around a mean value, but this appearance -on which other standard procedures such as min-max scaling depend upon- is not always correct, because the underlying function of the involved neural mechanisms could be related to other kind of waveforms that should require different treatment, so that the development of new methods that rather consider the characteristics of raw signals (lost sometimes after several layers of traditional filtering and averaging) to understand “rhythms” and their underlying functions is encouraged. To standardize the data, we did scale each of the EEG signals recorded to measure mental workload during execution of a specific task, for each subject in the dataset, for the theta frequency band, by means of universal scaling factors. We used universal scaling factors *f_theta_p*, and *f_theta_n* for samples with positive amplitudes, and negative amplitudes, respectively (as reference maximum and minimum values for the whole data set, to which local maximum and minimum values in each specific subset will be mapped or scaled), where *f_theta_p* = - *f_theta_n* (to reflect the symmetry between positive and negative amplitude values in the data). We also found the maximum and minimum values of amplitudes among all samples that belong (locally, or internally) to each specific subset (consisting of the data of one subject executing one of the tasks) and used these values as local scaling factors: (1) the maximum value for samples with positive amplitudes, and (2) the minimum value for samples with negative amplitudes in the subset. Then we mapped all the values in these samples from this local scale to the universal (MAX-MIN) scale. Let N, ℝ^+^, *S* = {*S*_1_, *S*_2_, …*S*_*s*_}, and *T* = {*T*_1_, *T*_2_, …*T*_*t*_} be the sets of natural numbers, positive real numbers, subjects in the dataset, and tasks in the dataset, respectively. Let *S*_*i*_, *T*_*j*_ = {*e*_*k*,_*S*__*i*_, *T*_*j*__|1 ≤ *k* ≤ *n*(*S*_*i*_, *T*_*j*_), *kϵℕ*} be the set of all trials of subject *S*_*i*_ executing task *T*_*j*_, after filtering through the theta band, and trimming, where each trial *e*_*k*,_*S*__*i*_, *T*_*j*__ is a set of samples, and let *n*(*S*), *n*(*T*), and *n*(*S*_*i*_, *T*_*j*_) be the number of elements of sets *S*, *T*, and *S*_*i*_, *T*_*j*_, respectively, where ∀*i*, 1 ≤ *i* ≤ *n*(*S*)[*n*(*S*_*i*_, *T*_6_) = 40, *n*(*S*_*i*_, *T*_1_)= 24, *n*(*S*_*i*_, *T*_2_) = *n*(*S*_*i*_, *T*_3_)= *n*(*S*_*i*_, *T*_4_)= *n*(*S*_*i*_, *T*_5_) = 20] are magnitudes known in the dataset after measurements. Let $e_{k,S_{i}{,T}_{j}}^{+}\subseteq e_{k,S_{i}{,T}_{j}}$, and $e_{k,S_{i}{,T}_{j}}^{-}\subseteq e_{k,S_{i}{,T}_{j}}$ be the sets of samples from trial *k*, having positive, and negative amplitude values, respectively.

To find the scaled version $\bar{S_{i},T_{j}}$ of subset *S*_*i*_, *T*_*j*_ we followed a three-step procedure:

First, we used universal scaling factors, defined by:


(1) $$\begin{array}{l} f\text{\_}theta\text{\_}p=usf,f\text{\_}theta\text{\_}n=-usf \end{array}$$

where *usf* = *780* in the present report, an optimized value used to set the universal MAX and MIN values to which the specific min and max values (or local scaling factors) of each subset *S*_*i*_, *T*_*j*_ are mapped. *f_theta_p* = *- f_theta_n* reflects the observed symmetry between the maximum positive values and minimum negative values in the datasets.

In this work we focus on subject dependent task transfer, but in general, we work in the implementation of universal classifiers, and also in the effect of scaling on classification results; therefore, in a more general context, we used *usf* as an independent variable, with *usf* taking any of 204 arbitrary values:


(2) $$\begin{array}{l} usf\in \left[1/1000,980\right]\subseteq {\mathbb{R}}^{+} \end{array}$$

The value *usf* = 780 was chosen because in the current data it already provides optimization in the classification results, simultaneously in both the theta band (that we consider in the present study), and the upper alpha band (that we also consider here to address some issues; see **Figure 3**).

Second, we found local scaling factors *local_f_theta_p*, and *local_f_theta_n* for subset *S*_*i*_, *T*_*j*_:

We found first the maximum values among all samples having positive voltage amplitudes, and the minimum values among all samples having negative amplitudes from each trial *k* in subset *S*_*i*_, *T*_*j*_:


(3) $$Max_{S_{i},T_{j}}=\overset{n(S_{i},T_{j})}{\underset{k=1}{\cup }}\max\left(e_{k,S_{i},T_{j}}^{+}\right)$$

(4) $$Min_{S_{i},T_{j}}=\overset{n(S_{i},T_{j})}{\underset{k=1}{\cup }}\min\left(e_{k,S_{i},T_{j}}^{-}\right)$$

From (3) and (4):


(5) $$\begin{array}{l} local\text{\_}f\text{\_}theta\text{\_}p=max\left(Max_{S_{i},T_{j}}\right) \end{array}$$

(6) $$\begin{array}{l} local\text{\_}f\text{\_}theta\text{\_}n=min\left(Min_{S_{i},T_{j}}\right) \end{array}$$

Third, we scaled all positive and negative samples *p* of each trial *e*_*k*,_*S*__*i*_, *T*_*j*__, using (1), and (2), together with (5), and (6) to define the set ${\bar{e}}_{k,S_{i},T_{j}}$ with the following equation:


(7) $${\bar{e}}_{k,S_{i},T_{j}}=\left\{\bar{p}|\bar{p}=\{\begin{array}{l} p^{*}f\_theta\_n/local\_f\_theta\_n,\;p\in e_{k,S_{i},T_{j}}^{-}\\ p^{*}f\_theta\_p/local\_f\_theta\_p,\;p\in e_{k,S_{i},T_{j}}^{+}\\ 0,p\in e_{k,S_{i},T_{j}},\;p=0 \end{array}\right\}$$

From (7):


(8) $$\begin{array}{l} \bar{S_{i},T_{j}}=\left\{{\bar{e}}_{k,S_{i},T_{j}}|1\leq k\leq n\left(S_{i},T_{j}\right),\;k\epsilon \mathbb{N}\right\}. \end{array}$$

**Figure 2:**
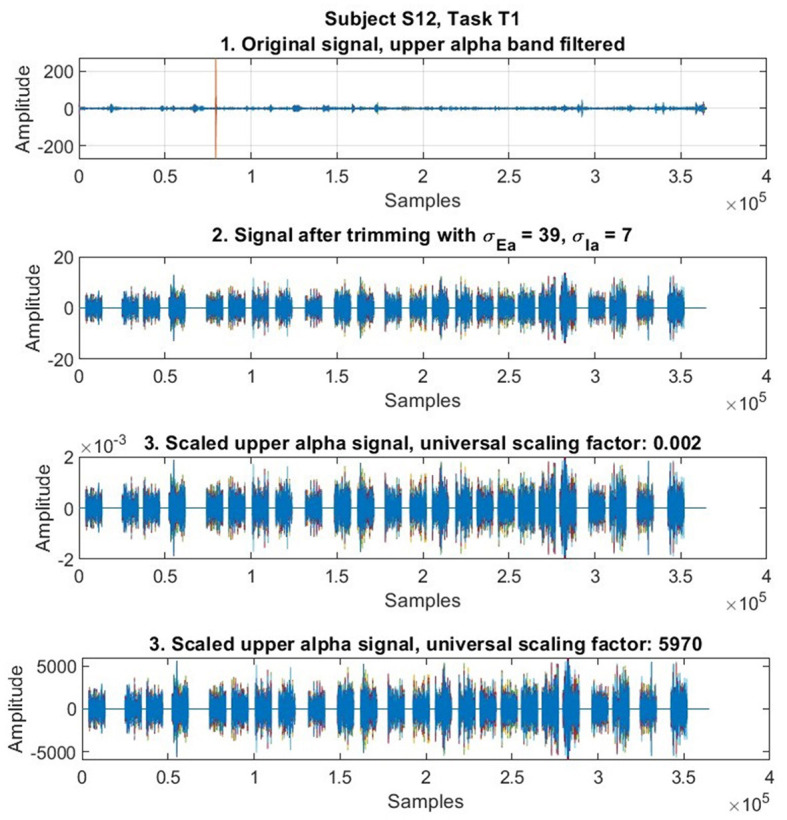
Effect of scaling. This procedure maps the amplitudes of the signals to scales determined by arbitrary universal scaling factors (usf). Here we illustrate the principle of scaling with a signal representing the execution of task T1 by subject S12 in the data by mapping the amplitudes of the original signal to two different arbitrary universal scaling factors (here 1/500, and 5970). As shown here, a usf can obviously be as arbitrary as one might want, but there are some practical limitations as to the number of them to be selected to run computer simulations. We ran 204 such simulations with positive universal scaling factors in the range (1/1000, 980), as indicated in Equation 2, that were sufficient to notice a tendence (Figure 3).

To summarize, for each trial in each data subset that belongs to a certain subject and task, our procedure maps all the positive amplitudes of the signals, based on the quotient between an arbitrarily set universal scaling factor (valid for all data subsets of subject-tasks) and the local maximum among all trails having positive amplitudes (local scaling factor) of the data subset at hand. And a similar procedure is done separately with the negative amplitudes. The effect of scaling according to the previous equations is illustrated in Figure 2.

**Figure 3:**
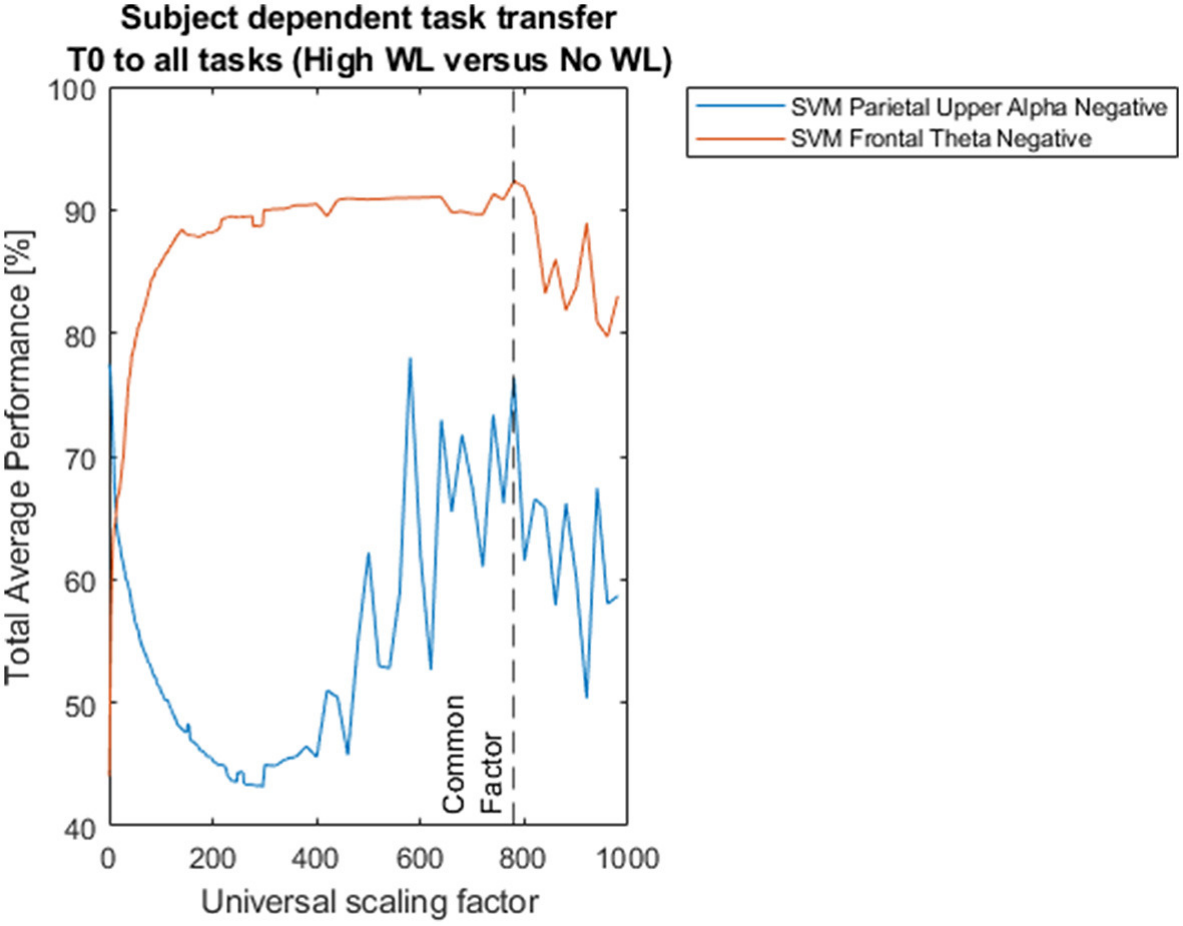
Impact of scaling on classification performances for subject dependent task transfer. Two hundred four universal scaling factors between 1/1000 and 998 were used in Equation 7 to scale all the subsets in the dataset to different sizes (signal amplitudes) after trimming, and before feature extraction and classification. All models of classification were based on support vector machines with radial basis functions and 5-fold cross-validation. The dotted black vertical line pinpoints total average performances of 92.35%, and 76.33%, corresponding to Support Vector Machine (SVM) classification between High Workload vs. No Workload, based on Frontal Theta Negative and Parietal Upper Alpha Negative oscillations feature extraction, respectively, for universal scaling factor f_theta_n = f_alpha_n = 780, common to both frequency bands.

In previous research, Shalabi et al. (2006) have reported that normalization of data through scaling is useful to improve classification results, particularly min-max normalization. In our case, which generalizes this approach, the rationale to adopt arbitrary universal scaling factors with Equations 1, 2 was to set these universal scaling factors as independent variables (with a total of 204 such values for such variables within the indicated domain in the presented data) in order to assess the impact of scaling on classification performance, together with the implementation of generalized classifiers of workload that are task and subject independent in further studies, that we have already started with promising results. Figure 3 shows the impact of different universal scaling factors on classification performance.

### 2.3 Feature extraction

**Figure 4:**
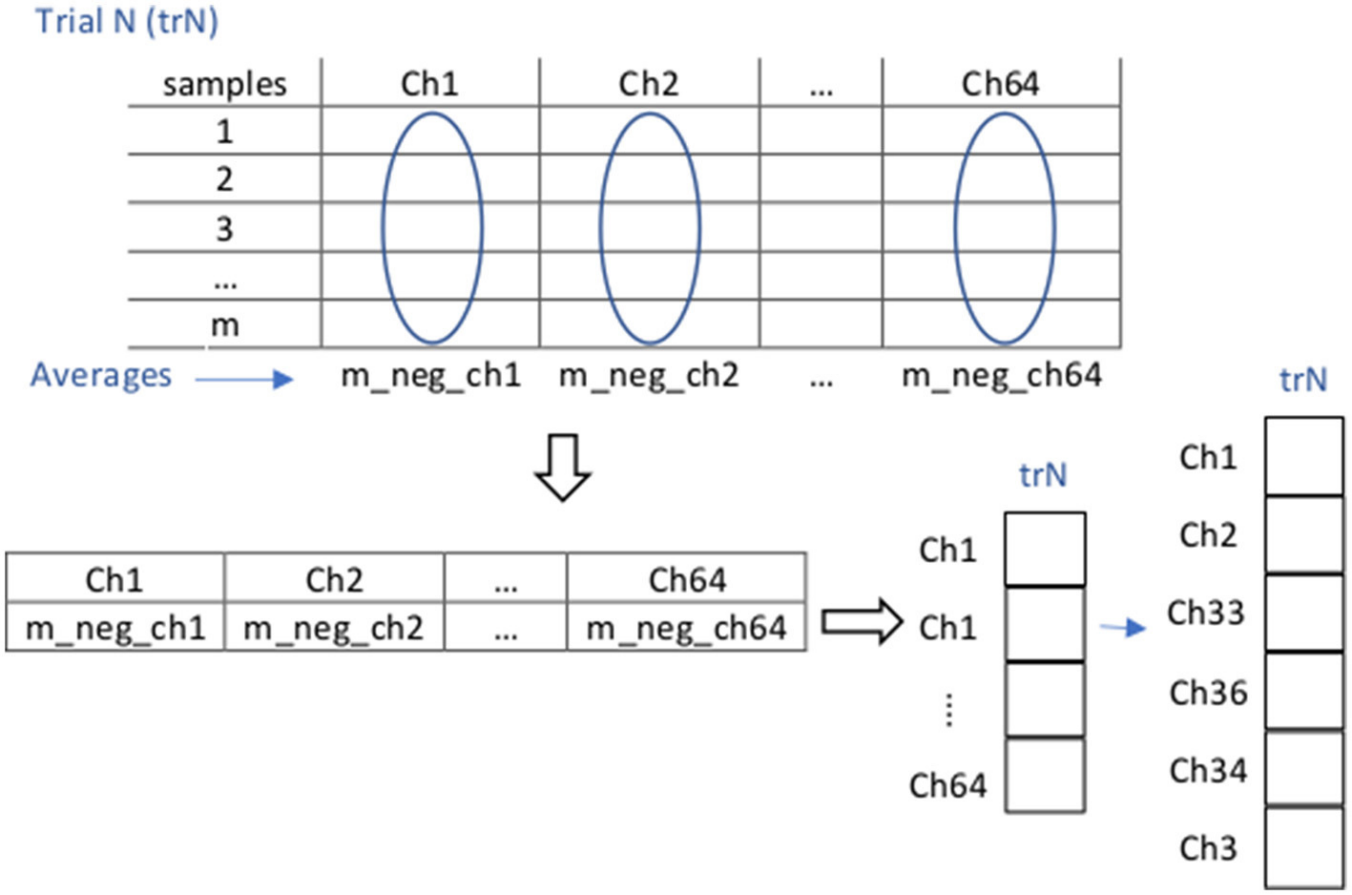
Illustration of the feature extraction procedure for negative Frontal Theta. For each N trial (trN) containing the data (a sequence of samples within the particular and whole timeframe of task execution, in all the 64 channels) corresponding to a specific subject and task, and that has already been preprocessed (in this case filtered through the theta band, trimmed, and scaled), feature extraction is implemented in two steps: (1) For each channel we average the negative components of all the samples of trial trN within the whole considered timeframe. As a result, we obtain a (64 × 1) dimensional vector representation of trN. (2) From this last vector, we extract data from channels ch1, ch2, ch33, ch36, ch34, and ch3 to obtain a (6 × 1) dimensional vector representation of trN.

We next extracted the negative components of these theta-filtered, trimmed, and scaled voltage values for further processing. After preprocessing, feature extraction for each trial was performed in two steps: First, for each trial (representing the execution of one of the tasks by one of the subjects), we found an average per EEG channel of all the sample components that have a negative voltage amplitude in that channel. The average of each trial reflects at the end an average over the duration of the task it represents: we took all the samples present in the whole timeframes considered for each of the tasks (T0: 9 s, T1: 20 s, T2: 24 s, T3-T5: 30 s). As a result of these averages, each trial was represented by a feature vector of dimension 64 × 1 (our signals consist of 64 channels). Second, among these 64 channels we selected the six channels that exhibited the largest change in theta negativity (the absolute negative highest average voltages), according to a previous analysis of the whole dataset (across all subjects), which resulted in a 6-dimensional feature vector for each trial, reflecting frontal theta negativity. These six channels were ch1, ch2, ch33, ch36, ch34, and ch3, that according to the extended 10-20 system layout for the actiCAP 64ch correspond to EEG electrodes FP1, FP2, AF7, AF8, AF3, and F7, respectively, located on the brain prefrontal area. After this, each trial was represented by a feature vector of dimension 6 × 1 (Figure 4).

**Figure 5:**
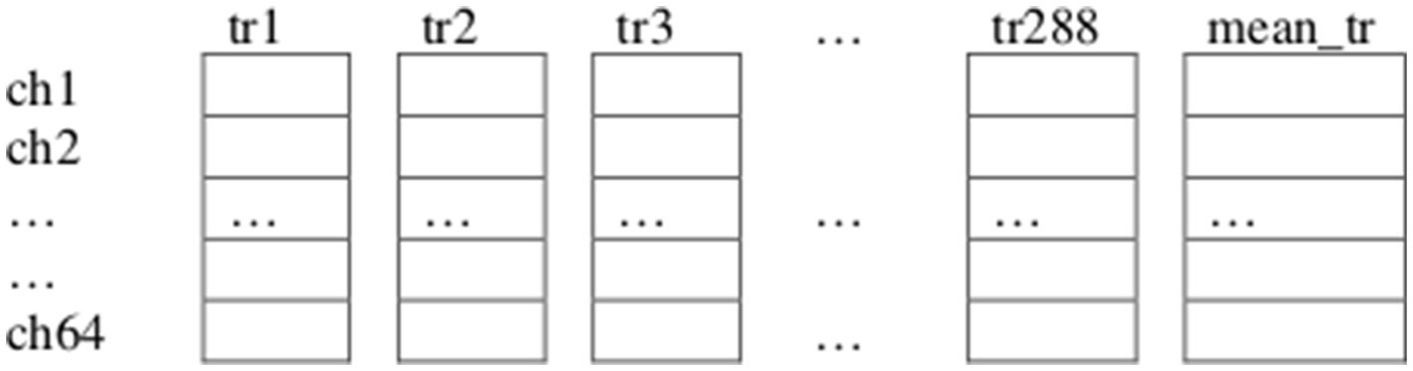
Illustration of the “Theta Negative” Matrix TN (64 × 288), and vector mean_tr (64 × 1) corresponding to the total 288 trials of task T1. Collected from all the twelve subjects considered for analysis from our dataset (each subject performed 24 trials of task T1), each trial (previously preprocessed, filtered through the theta frequency band, trimmed and scaled) is represented by a vector (64 × 1), obtained after finding an average per EEG channel of all the sample components that had a negative amplitude in that channel.

To determine channels ch1, ch2, ch33, ch36, ch34, and ch3 as those to be considered for feature extraction we proceeded as follows, for each of the tasks in our paradigm: First, we repeated the previously explained first step for each of the trials of all the subjects in the dataset for the current task. Second, we collected all the resulting averages for each of these trials in a “Theta Negative” matrix TN (num_channels × num_trials) where num_trials is the total number of trials from all subjects executing the current task (Figure 5). Third, we computed a vector mean_tr (num_channels × 1), which is the mean of Matrix TN across columns (trials). Fourth, we identified the channels containing the n_highest (n_highest = 6) highest absolute negative values in vector mean_tr.

**Figure 6:**
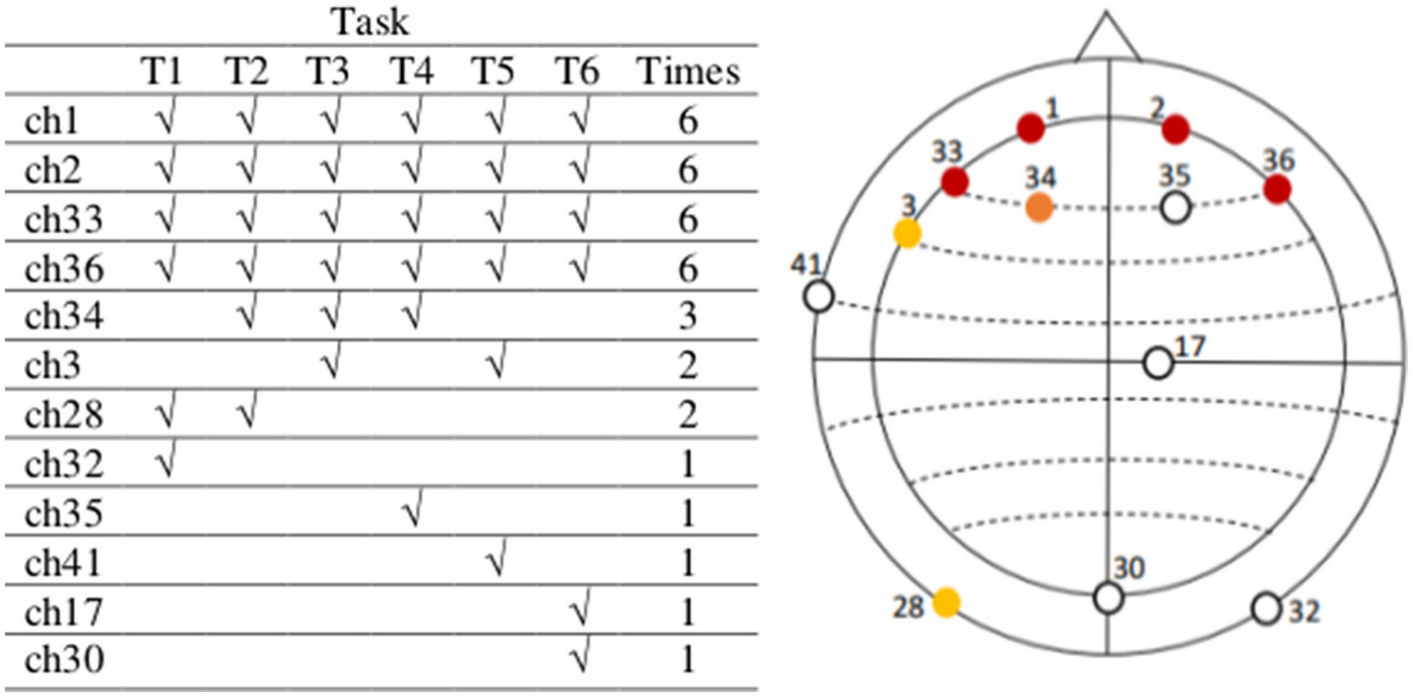
The most active EEG channels with data in the theta frequency band are in the pre-frontal and frontal region, related to mental workload. The most active EEG channels during task execution, after an average analysis per task, considering the data of all subjects in the theta frequency band are channels 1, 2, 33, 36, 34, and 3, located on FP1, FP2, AF7, AF8, AF3, and F7, respectively (actiCAP 64Ch layout). After an independent average over each task these are the most active channels in all tasks. We use the information in these six channels in the second step of our feature extraction method. Note that there are some channels out of the relevant topographic region (among the least common, except for ch35), but these are not active beyond one single task. Between channels ch3 and ch28, we have selected ch3 because it is nearest to those in the most active (or populated) region.

The resulting channels containing the six highest absolute negative values in the vectors mean_tr obtained after performing the previous four steps for each of the six tasks in our data are indicated in Figure 6 (table on the left) and depicted identified in their corresponding electrode positions according to the actiCAP 64Ch layout in the same figure (right). Finally, we selected those channels that characterize the activities of tasks the most times. For instance, we see Figure 6, that channel ch1 repeats six times, which means that ch1 was always one of the top six channels containing the highest absolute negative alpha amplitude values in the vectors mean_tr in all the six tasks. The selected EEG channels are: ch1 (FP1), ch2 (FP2), ch33 (AF7), ch36 (AF8), ch34 (AF3), and ch3 (F7).

**Figure 7:**
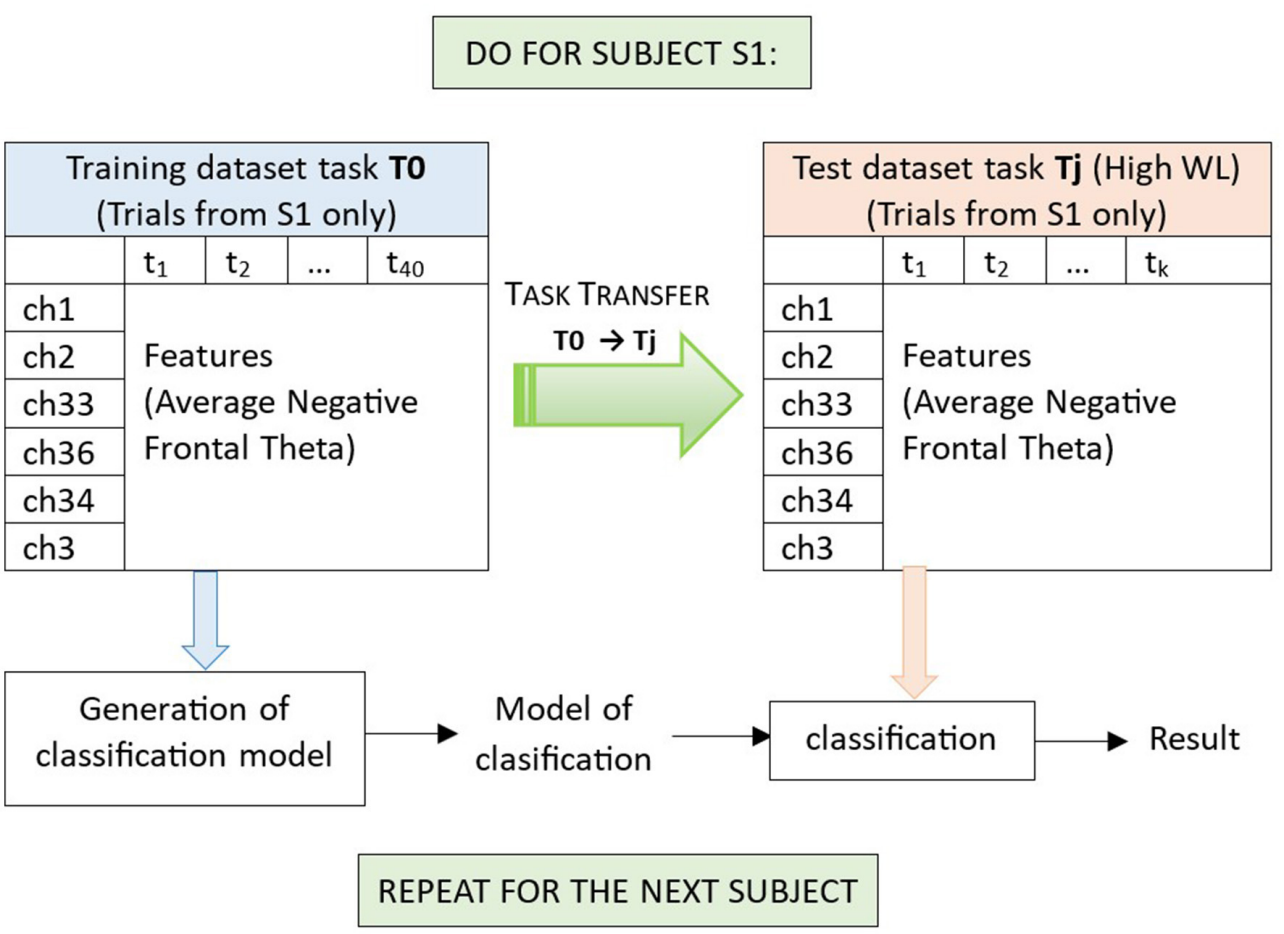
Illustration of the generation of a model of classification, and classification in subject dependent task transfer from task T0 to a task Tj (with data of subject S1). In negative Frontal Theta Subject Dependent Task Transfer, trials have been filtered through the theta frequency band, and obtained after negative Frontal Theta feature extraction (section 2.3, in which a leave one out analysis for subject S1, information from EEG channels 1, 2, 33, 36, 34, and 3 is retained). We use T0 as training dataset (which contains 20 trials of the no workload condition and 20 trials of a workload condition for subject S1). For training, we use Support Vector Machines with radial basis functions and five-fold cross-validation (per condition we have five training experiments, each reserving 16 trials for training and four trails for testing). Once a model of classification is obtained for T0 (subject S1), we test this model to classify the trials of another task Tj, with data from the same subject S1 and one of the two workload conditions. Here, task Tj is used as testing dataset, and contains k trials of the high workload condition, collected from the same subject S1. Finally, average performances per task across all subjects are obtained (Tables 1, 2).

We have included the data of all subjects to illustrate the feature extraction procedure that we use but, in each iteration of task transfer, from the data of all subjects we will subtract the data of the current subject to determine the feature channels to be used to process the data of this subject, to avoid any concerns about data leakage. In this work we perform subject dependent task transfer, from T0 to T1-T5, which means that for each subject Si we will train a model of classification (with cross-validation) on data from T0, and then test this model on the (unseen) data from T1-T5 for the same subject Si, and then repeat the procedure for the next subject (see also Figure 7). For each of these iterations per current subject Si, the feature channels will be determined by the procedure already described, considering the data of all subjects with exemption of Si. In this way, we also manage to keep a general tendency we are looking for in the data (EEG channels related to mental workload, ideally stable) by using the largest possible amount of data, but at the same time we guarantee that in each iteration we use a model of features with data unseen for a new subject that did not participate in their determination.

Our feature extraction method relies on a general tendency that only appears by averaging large amounts of data belonging, ideally, to the largest possible number of subjects (and or tasks according to the case) to train the data. The literature of EEG has previously presented cases in which features are only observable after using grand averages of data, such as in the case of event related potentials (Hoehl and Wahl, 2012; Voigt-Antons et al., 2012; Kuncheva and Rodríguez, 2013), also including specific cognitive tasks such as n-back (Aksoy et al., 2021) and the readiness potential (“Bereitschaftspotential”) (Blankertz et al., 2007; Nann et al., 2019), where grand averages have a noise canceling effect in the signals, and some of these event related potentials, occurring at longer latencies after stimuli are more related to endogenous brain states (Nunez, 2012; Sellers et al., 2012), like the ones we are looking for. We have presented a simple classification method with channel selection based on a general tendency in the data obtained along the whole durations of trials which should relate mental workload to a more permanent or endogenous characteristic of mental activity.

The choice for negative over positive samples is essentially arbitrary, and they both work very well, as we have shown (see Section 4) with very high classification performances and no significant difference between these two groups. Also, having in mind that our first objective was to improve classification performances previously obtained with other disseminated methods, we considered that whatever the case (either negative or positive samples) we needed a rather new feature representative of the EEG brain activity, and that if we used a single feature per EEG channel, we would already have a 1 × 64 dimensional feature vector per trial, so we used a very simple feature, namely the average of the amplitudes of the signals in each channel, and then carried out dimensionality and data reduction with feature extraction already described in this section.

### 2.4 Classification

We classified the extracted 6-dimensional feature vectors with a support vector machine (SVM) with radial basis functions, trained with five-fold cross-validation. For each subject, the classifier was trained on all trials from the calibration task (T0). Then, the classifier was separately tested on all trials from each other task (T1-T5). MATLAB was used to implement the analyses, with its Statistics and Machine Learning Toolbox for SVM classification.

Figure 7 illustrates the classification procedure for high workload vs. no workload for subject dependent task transfer from T0 to a task Tj (T1-T5). For T0, a five-fold cross-validation was used to estimate the classification accuracy on T0 itself and to generate a model of classification, tested on T1-T5, respectively.

## 3 Results

**Figure 8:**
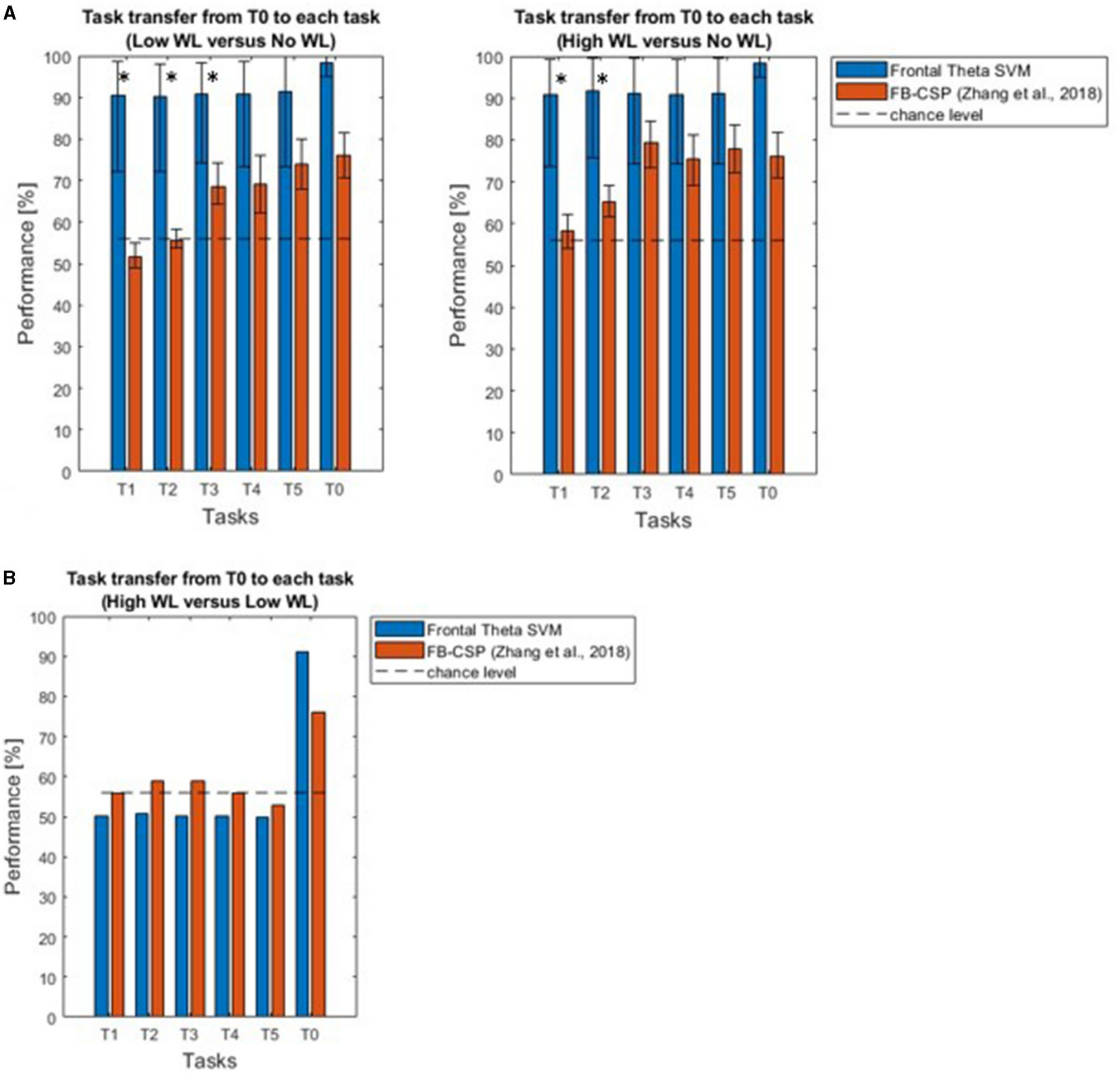
**(A)** Support vector machine based on frontal theta oscillation features enables classification of high and low mental workload vs. no workload across tasks. Across different tasks (T1-T5) used for testing the workload classifier trained on the calibration task (T0), the existing approach based on a filter-bank common spatial patterns algorithm failed to achieve above chance-level performance. Our novel approach based on a support vector machine outperforms this classifier, allowing for robust across-task classification of workload in T1 and T2. Asterisks indicate where performance was higher using our approach, compared to that of Zhang et al. (2018) (Wilcoxon signed-rank test, *p* < 0.05, Bonferroni corrected). **(B)** Results with our methodology (with an average performance of 57.04% across tasks) did not go beyond chance level and remained below the performance of the previous approach (59%) for task transfer classification between high vs. low workload from T0 to T1-T5.

We found that our approach outperformed the inconsistent performance of Zhang et al. (2018) (Table 1, Figure 8A), when comparing high (or low) mental workload with no workload. Performance significantly deviated from chancel-level (Wilcoxon signed-rank test, *p* < 0.05, Bonferroni corrected) in each of T1-T5. Furthermore, the performance of our approach was significantly higher than that of Zhang et al. (2018) in T1 and T2 (Wilcoxon signed-rank test, *p* < 0.05, Bonferroni corrected).

**Table 1:** Support vector machine based on frontal theta oscillation features enables classification of mental workload across tasks.

**Performances (%)** (Mean ± SD)^*^	**T1 N-Back**	**T2 Span**	**T3 Add**	**T4 Word**	**T5 Rotation**	**T0 (calibration)** **Subtraction**	**Average**
**Low vs. no workload**							
Zhang et al. (2018)	52 ± 6	56 ± 4	68 ± 10	69 ± 14	74 ± 13	76 ± 11	65.83
Frontal Theta SVM	90.56 ± 20	90.17 ± 19	90.83 ± 19	90.67 ± 20	91.50 ± 20	98.25 ± 4	92.00
**High vs. no workload**							
Zhang et al. (2018)	58 ± 8	65 ± 7	79 ± 11	75 ± 13	78 ± 12	76 ± 11	71.83
Frontal Theta SVM	90.83 ± 20	91.83 ± 18	91.17 ± 20	90.83 ± 20	91.17 ± 20	98.25 ± 4	92.35

Across different tasks (T1-T5) used for testing the workload classifier trained on the calibration task (T0), the existing approach based on a filter-bank common spatial patterns algorithm failed to achieve above chance-level performance. Our novel approach based on a support vector machine with features from frontal theta oscillations outperforms this classifier in T1 and T2, allowing for robust across-task classification of workload. These results correspond to a common universal factor usf = 780 (Figure 3).

^*^Across Subjects.

On the other hand, for classification between “high” vs. “low” workload conditions our Support Vector Machine based on Frontal Theta Oscillations reached only an average performance of 57.04% for task transfer from the “calibration” task to all tasks, not beyond chance level, and lower than the 59% performance of the previous study (Figure 8B).

## 4 Discussion

We have introduced an approach allowing for classification of mental workload from EEG signals across five tasks commonly employed in the field. Our approach is based on the extraction of theta negativity from frontal EEG sensors, which is then fed into a SVM classifier, in contrast to the prior approach based on the FB-CSP classifier.

While existing signal processing approaches usually assume that brain oscillations are sinusoidal, this assumption has been found to be false in numerous recent studies (Cole and Voytek, 2016). This may occur when the negative portion of an oscillation exhibits behavior distinct from the positive portion. For this reason, instantaneous voltage has been proposed as an alternative to power and phase-based interpretation of oscillatory brain activity (Schalk et al., 2017). In the spirit of this proposal, we consider only the amplitude of the negative portion of oscillatory activity in the theta band and show that it can lead to superior performance over traditional power-based features in some comparisons.

Our trimming procedure treated all data equally and automatically, with parameters that not only neutralize extreme abnormal values that exceed a certain threshold of standard deviations in each dataset per subject and task, but that also are optimized so that they do not affect original data that should not change. After the application of the trimming procedure an estimated 98.37% of all samples in the whole dataset remained intact. We verified that trimming had no noticeable effect on classification performance, but we realized its importance to our feature extraction procedure: it deemphasized the magnitude of aberrant voltage values in contaminated channels that otherwise would have wrongly been considered as the activated source of involved brain activity. It would not have made sense either to scale the data if we had not first removed these aberrant values.

The guiding objective behind the adoption of our general scaling procedure is the implementation of universal (both task and subject independent) classifiers of workload. In the previous scaling equations, while local scaling factors are inherent to each subset to be scaled -local max and min values-, universal scaling factors *f_theta_n*, and *f_alpha_n*, determine the ranges between unique and general maximum and minimum values to which all subsets will be mapped before further meaningful comparisons between data from different subjects and tasks -also originated from different recordings- in the classification step are made.

And, as we have seen (Figure 3) scaling has an impact on classification performance. The classification accuracies are dependent on the absolute value of the universal scaling factors because the radial basis function (RBF) kernel we use for the SVM is non-linear. Here, scaling the feature space affects the kernel width and this translates into different classification output. We verified that the sensitivity is solely the effect of the kernel, and to double-check we replaced the kernel with a linear one, and also tried other linear options, and this does not make the classification output dependent on the scaling, but it rather stabilizes into, or within, clear constant linear tendencies.

As to the question of what would happen with classification results if samples with positive amplitudes were used instead of those with negative amplitudes, we conducted an analogous procedure of preprocessing, feature extraction (with the same leave one out analysis), and classification, but this time based on positive values instead of negative values of signals in the theta-band, with high classification performances: 80.22% for Frontal Theta SVM based on positive values, vs. 90.64% based on negative values, with no significant difference between these two groups (tested with the Wilcoxon signed-rank test, *p* < 0.05, Bonferroni corrected in each of T1-T5) in low vs. no workload; and 80.10% for Frontal Theta SVM based on positive values, vs. 91.27% based on negative values, also with no significant difference between these two groups (tested with the Wilcoxon signed-rank test, *p* < 0.05, Bonferroni corrected in each of T1-T5) in high vs. no workload, with the same universal scaling factor 740 for all cases.

**Figure 9:**
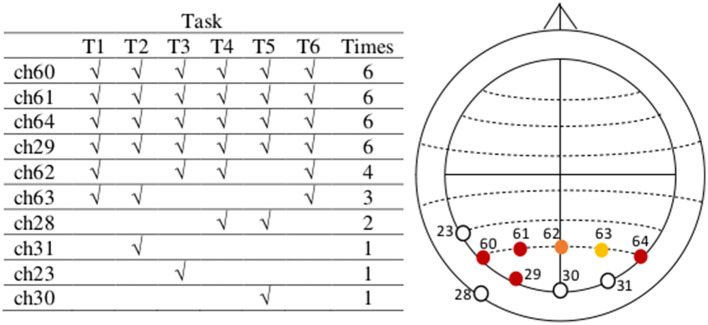
The most active EEG channels with data in the upper alpha frequency band during task execution are in the parietal, and parietal-occipital region, related to mental workload. After filtering the same data through the upper alpha band (10–13Hz) and following an analogous procedure of preprocessing and feature extraction (Sections 2.2. and 2.3), considering the data of all tasks and subjects, the most active channels are 60, 61, 64, 29, 62, and 63, located on PO7, PO3, PO8, O1, POz, and PO4, respectively (actiCAP 64Ch layout). All channels are in the neighborhood of the most active region.

It could be said that Figure 6 presents channels where eye blinks are recorded. At the same time, they are channels on locations physiologically associated with mental workload activity in the theta-band. This led us to investigate and check this question: if, as an alternative, the same data were filtered in the upper alpha band (10-13 Hz) for instance, and an analogous procedure applied, which brain regions will be activated if any mental workload occurred? To be sure about this, we implemented such analogous procedure with the same data filtered this time through the upper alpha band, and our methods pinpoint the parietal and parietal-occipital regions as the most active (Figure 9), corroborating previous findings in the literature regarding this frequency band and mental workload (Gevins et al., 1997; Stipacek et al., 2003; Gerjets et al., 2014). In both frequency bands, our combination of preprocessing and feature extraction methods applied to the data finds regions associated to mental workload in these respective bands. This analysis does not rule out any influence of eye activity (see below for our analysis addressing eye blinks more directly with independent component analysis ICA) but does point to the general flexibility of this method in finding relevant electrode sites in line with hypotheses.

**Figure 10:**
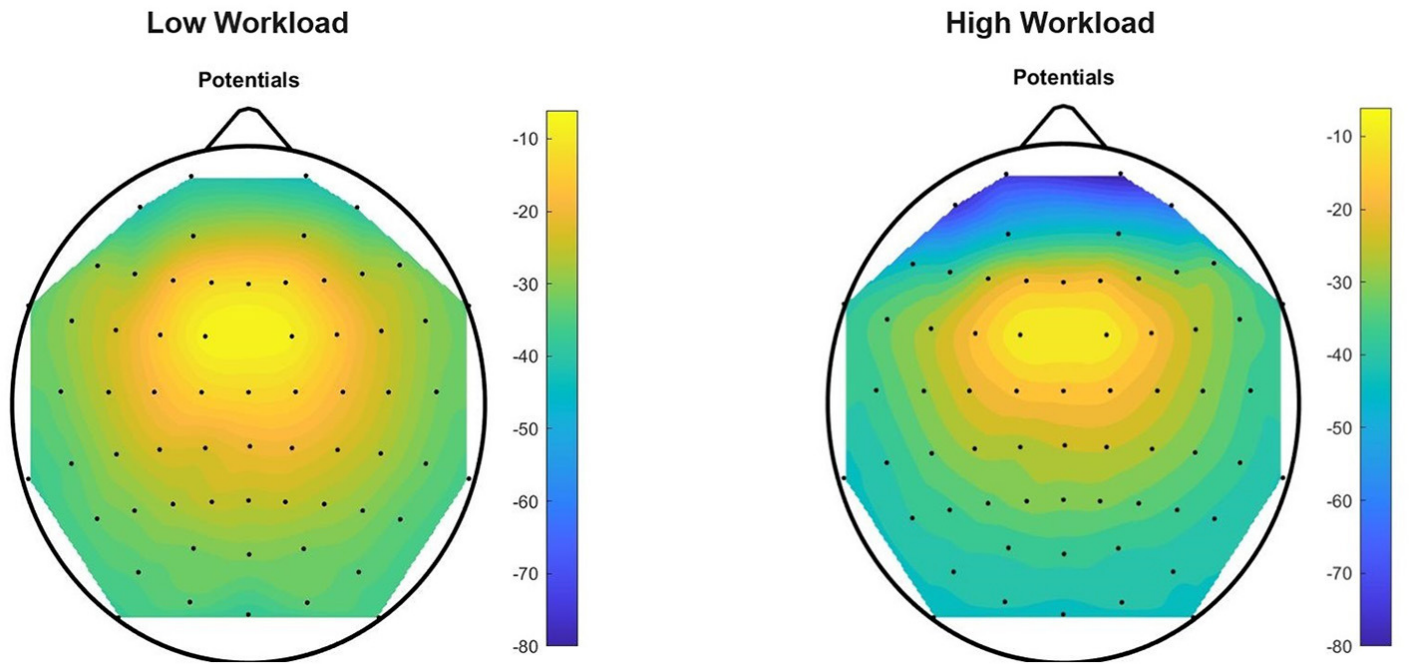
Theta Negative topographic colored maps over the 64-channel actiCAP layout of the means of vectors (1 × 64) of trials performed by all the 12 subjects, for each of the two workload conditions, for task T0. For each trial (preprocessed with theta band filtering, trimming and scaling with Equations 1–8) we found an average per EEG channel of all the sample components of the trial that have a negative amplitude in that channel. Afterwards, for each task, we computed an average of these mean values [vector representations (1 × 64) of trials] across all the subjects in the dataset.

**Figure 11:**
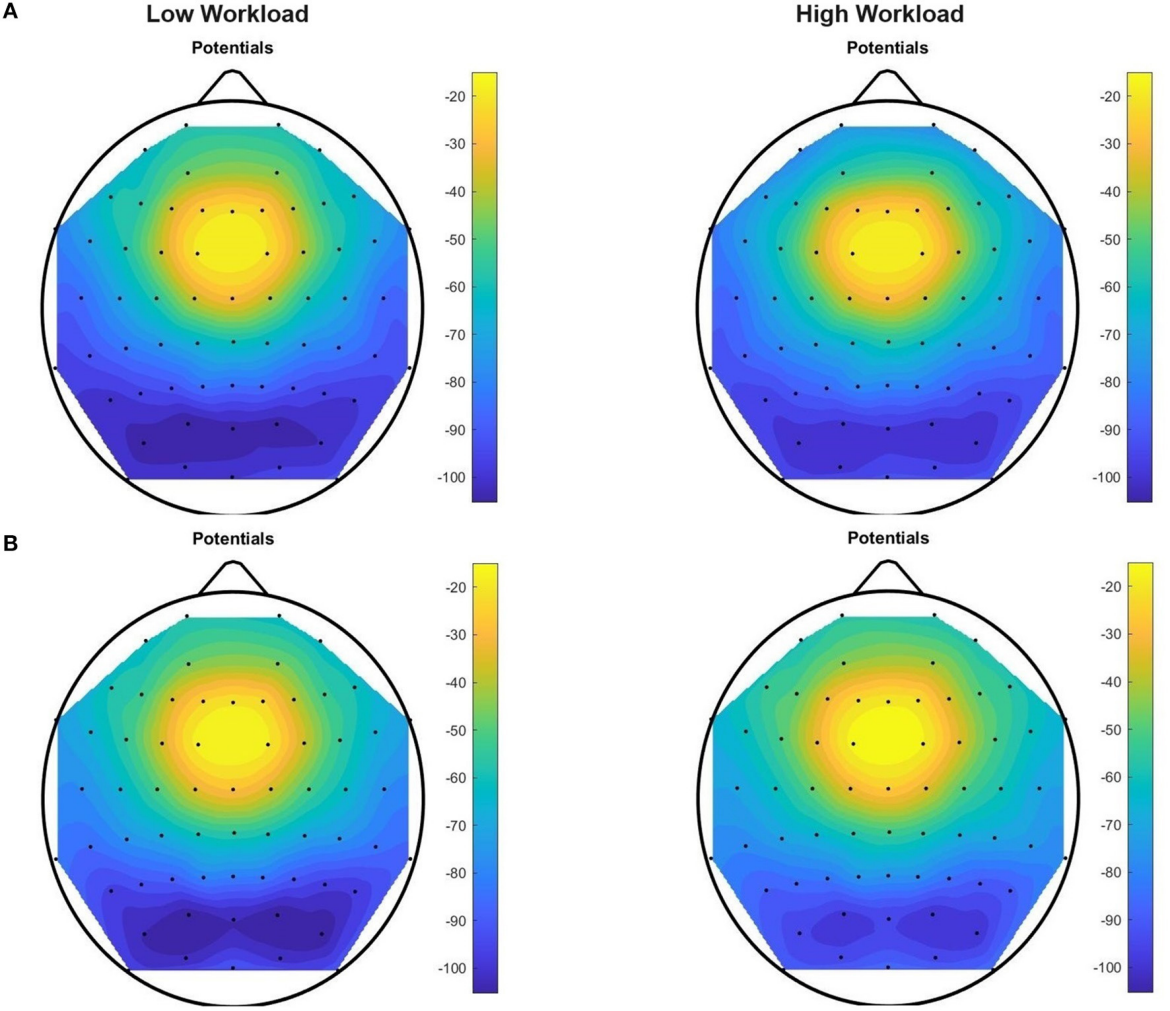
Upper Alpha Negative topographic colored maps over the 64-channel actiCAP layout of the means of vectors (1 × 64) of trials performed by all the 12 subjects, for each of the two workload conditions, for tasks T0 **(A)** and T1 **(B)**. For each trial (preprocessed with upper alpha band filtering, trimming and scaling with Equations 1–8) we found an average per EEG channel of all the sample components of the trial that have a negative amplitude in that channel. Afterwards, for each task, we computed an average of these mean values [vector representations (1 × 64) of trials] across all the subjects in the dataset.

We have determined (Figures 10, 11) that the most active EEG electrodes during task execution are those in the frontal and pre-frontal regions (FP1, FP2, AF7, AF8, AF3, and F7) for data in the theta band, and in the parietal and parietal-occipital regions (PO7, PO3, PO8, O1, POz, and PO4) for data in the upper alpha band. This finding based on data from all subjects was consistent in all tasks and we used information in these most active electrodes for feature extraction. After computing our six “Theta Negative” (TN) Matrices (Figure 5), for each of the six tasks of the paradigm, and their mean values across columns [vector representations (1 × 64) of trials collected from all the subjects in the dataset] we plotted a topographic colored map with these mean values for each of the two workload conditions present in the data (Figure 10). Similarly, for the upper alpha frequency band, we computed six “Alpha negative” (AN) Matrices and their mean values across columns and plotted a topographic colored map with these mean values for each of the two workload conditions (Figure 11).

Evidence that we are dealing with mental workload is highlighted after inspection of these topographic colored maps. Figure 10 confirms an increase in theta absolute negative amplitudes for EEG activity in the frontal and pre-frontal brain regions, as workload increases (in T0, from the “no” workload condition to the “workload” condition), and Figure 11 confirms a decrease in alpha absolute negative amplitudes for EEG activity in the parietal-occipital, and parietal brain regions as workload increases, per task (from the “low” to the “high” workload condition) in both T0 and T1, and for each of the two conditions, as measurements continue from T0 to T1 (corroborated also through T2, T3, T4, and T5, though visual data of these tasks are not shown in the current figure).

As a verification step we removed eye activity with independent component analysis. To apply ICA cleaning, we followed (Klug and Gramann, 2021) for preprocessing, where all data from one participant was concatenated, subsampled to 250 Hz, and high-pass filtered using a Hamming windowed sinc FIR filter with a passband edge at 1 Hz (−6 dB cutoff at 0.5 Hz) before applying AMICA (Palmer et al., 2012) using the EEGLAB plugin (v1.7) with automatic cleaning enabled (five rejections at three standard deviations). Weights were then copied back to the original, individual task datasets, and independent components (ICs) were classified using ICLabel (v1.3) (Pion-Tonachini et al., 2019). Finally, ICs for which the probability of “eye” was higher than any other type were removed from the data. Afterwards, we took the data through the pipeline consisting of preprocessing, feature extraction, and classification, as previously described.

This leads us to one limitation: in spite of the fact that we took care to remove artifacts with our trimming procedure in the preprocessing phase, prior to feature extraction, classification, and future analyses, that effectively removed such abnormal, out of range values, while keeping an estimated 98.37% of the data intact, and from the fact that 1.63% of the whole dataset was affected by higher amplitude peaks, so that ocular artifacts must account up to such percentage of the data at most and likely procedures to remove such artifacts should not affect more than such 1.63% of the data (thus have a big impact on classification performances), and that additionally, as detailed above, after applying our procedures to the same data in the upper alpha frequency band we found once again the most activity in channels which according to the literature are associated to mental workload, after conducting the ICA to remove artifacts from the original data (before any further procedures, including preprocessing), the classification performances for subject dependent task transfer dropped as shown in Table 2.

**Table 2:** Frontal theta oscillation features classification of mental workload across tasks after independent component analysis (ICA).

**Performances (%)**	**T1 N-Back**	**T2 Span**	**T3 Add**	**T4 Word**	**T5 Rotation**	**T0 (calibration)** **Subtraction**	**Average**
**Low vs. no workload**							
Zhang et al. (2018)	52	56	68	69	74	76	65.83
ICA Frontal Theta SVM	66.95	68.83	64.17	65.33	66.83	95.67	71.30
**High vs. no workload**							
Zhang et al. (2018)	58	65	79	75	78	76	71.83
ICA Frontal Theta SVM	67.50	66.17	66	63.33	64.17	95.67	70.47

After initial treatment of the datasets with ICA, previous to our same described pipeline of preprocessing, feature extraction, and classification across different tasks (T1-T5) used for testing the workload classifier trained on the calibration task (T0), our approach did not outperform the existing approach based on a filter-bank common spatial patterns algorithm, but nevertheless achieved more stable and uniform performances above chance-level for all tasks. A common universal factor usf = 680 for both (the Upper Alpha and the Theta Band) was used in this case, for data treated with ICA.

^*^Across Subjects.

**Figure 12:**
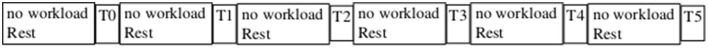
Alternate sequence of task execution for the current experimental paradigm. A consistent introduction of a “no” workload (“rest”) condition between all task blocks will attenuate the effect of cumulative “mental load” for blocks of tasks executed later in time, enabling a more precise estimation of workload strictly due to the tasks themselves.

For future work it also remains the question of levels of mental load due to execution of tasks themselves. One possibility should be the introduction of a “no” workload (“rest”) condition consistently between all the different blocks of task execution (Figure 12), instead of only one block in T0 at the beginning, as it is possible that current findings are at least in part due to the effects of fatigue or other non-stationarities that inevitably influence EEG activity over time. In the current scenario it remains the question of whether subjects undergo a cumulative effect of “mental load” as time elapses and further blocks of tasks are continuously executed with no “rest” in between, that must be contributing to the “mental load” strictly due to the tasks, with an increasing effect in tasks executed at later points, furthermore when no randomization in the order of task blocks exists. This may help explain why in (Zhang et al., 2018) when comparing a workload condition with rest, “notably n-back and backward span tasks, appear to consistently elude reliable classification,” while add, word, and rotation (executed later) had better classification performances, whereas in the comparison between “high” vs. “low” workload conditions such cumulative effect was absent in that work.

Also, the introduction of a “no” workload condition in-between tasks would be beneficial to generalize the analyses and results to a more Universal Workload Classification. Due to the absence of the “no” workload condition in all the tasks, except for T0, we only analyzed task transfer with classification between “no” workload and a workload condition, from T0 to T1-T5, but such inclusion will enable analysis of task transfer from any task to other tasks, with trials from all subjects.

Nevertheless, the current (or a similar) basic sequence, consisting of a “rest” condition only for a task at the beginning, followed by execution of several tasks should suit studies where the investigated variable is “mental workload” *per se*, increasing independently from task nature that in such case will not be as relevant as the question that as time elapses so must mental ‘load' increase, allowing possibilities to measure workload because at the end of every measurement we will be at the end of a scale in comparison to the initial point in time (the “rest” condition), and raising other questions: which brain areas are the most active at the beginning (minimum load), in transition, and at the end (maximum load)? Are these most active regions independent from the selected tasks, and therefore, does mental workload activate specific regions consistently?

Another limitation is that our approach shows lower performances than the previous approach when comparing high vs. low workload conditions. This lack of success in contraposition to the high performances to discriminate between a workload condition (either “high” or “low”) vs. a rest condition, suggest improvements not only to classification algorithms, but also to methods that assign degrees of difficulty to paradigmatic tasks. This however was beyond the reach of the present study.

The approach investigated here adds one more tool to the myriad options to classify workload in various conditions; specifically, a computationally lightweight method that can be a better choice depending on the requirements of the scenario, such as detection of mental workload. This can serve several real-world applications, such as detecting fatigue in safety-critical situations (Monteiro et al., 2019). After a brief training session, a BCI could be employed to detect changes in mental workload across a variety of tasks that the user could engage in, without the need for retraining the system.

## Data availability statement

The original contributions presented in the study are included in the article/Supplementary material, further inquiries can be directed to the corresponding author.

## Ethics statement

Ethical approval was not required in accordance with local legislation and institutional requirements. The participants provided their written informed consent to participate in the study.

## Author contributions

GGA developed the methods, created models of data, implemented computer programs, performed the formal analysis, wrote the report, prepared the visualization and data presentation, and generated and annotated data and code for reuse and replication. DH selected the tools for statistical analysis, wrote the report, and performed critical review, including major revisions. LRK conceptualized the project and its paradigm, supervised the experiments, data collection, and the development of the project, performed an Independent Component Analysis (ICA), and provided critical review, editions, and major revisions to the manuscript. SRS administered the project, oversighted the execution and development of the research activities, and provided critical review, and revisions to the manuscript. TOZ conceptualized the central ideas, goals and objectives of the project, provided the data, and oversighted the execution and development of the research activities. All authors contributed to the article and approved the submitted version.

## Supplementary material

The UniWC_Toolbox_v9 for Universal Classification of Mental Workload, a fully documented computer software in Matlab under the GNU Public License, to replicate all the results and figures in this paper, as well as all intermediate data, is openly and publicly available at ZENODO with DOI: 10.5281/zenodo.10077137.
